# Utilising Palliative Care Expertise in Critically Ill Patients: Opportunities to Improve Outcomes and Experiences

**DOI:** 10.3390/jcm14124275

**Published:** 2025-06-16

**Authors:** Tajveer Sara, David Walker

**Affiliations:** Department of Critical Care, University College London Hospital, London NW1 2BU, UK; d.walker@ucl.ac.uk

**Keywords:** palliative care, intensive care, trigger tools

## Abstract

**Background**: Prior to the COVID-19 pandemic, the mortality of patients admitted to intensive care units (ICUs) in the UK reached 20%. Studies show up to 40% of these patients may have been eligible for palliative care (PC) referral on admission to the ICU. The involvement of PC teams improves the quality of care delivered and improves patient and family satisfaction. Several trigger tools have been developed to identify ICU patients most likely to benefit from palliative care; however, no consensus exists regarding the most effective tool. **Methods**: We conducted a retrospective study to identify the number of PC referrals, prior to death in a general ICU setting over a 7-month period in 2019. For patients not referred to PC, three separate “trigger tools”, previously described in the literature, were retrospectively applied to explore the potential impact each tool may potentially have had on PC referral rates. **Results**: We identified 121 ICU deaths, of which 28 patients (23%) were referred to PC during their admission to the ICU. After retrospective application of the trigger tools to those who were not referred, 75% (n = 70) of patients triggered at least one criterion using the “Zalenski et al.” tool and 71% (n = 66) of patients were eligible for referral had the “Hua et al.” tool had been used. Overall, 82% (n = 36) of cancer patients met at least one criterion for referral with the Royal Marsden tool. **Conclusions**: Our study supports a finding of poor utilisation of PC services in an ICU patient population. The use of trigger tools can be used to significantly increase the number of appropriate PC referrals in an intensive care setting and may be useful in predicting those who may die. The benefit of PC intervention is aimed at providing holistic support to both patient and family and is associated with better patient and family experience towards the end of life.

## 1. Introduction

### 1.1. Dying in Hospital

Over the last century, medical advancements and increased life expectancy have shifted societal expectations around illness, longevity, and death. In 1900, life expectancy was approximately 45 years; by 2021, it had risen to 81 years [1]. This has influenced the timing, causes, and location of death [2]. With the establishment of the National Health Service (NHS) in the UK in 1948 and increased access to a growing range of medical and surgical interventions, more people are now dying in hospitals. Despite surveys indicating a preference for home deaths [3], nearly 50% of deaths in England and Wales in 2016 occurred in hospitals [1], with around 35% of inpatients recognised as being in their last year of life [3].

Chronic diseases often follow a pattern of relapses and remissions, with hospital admissions increasing in the final year of life [4]. The significant number of hospital deaths highlights two important issues in end-of-life care (EOLC). Firstly, patients may inevitably lack autonomy or capacity in making EOLC decisions and secondly healthcare providers may struggle to predict disease trajectories with accuracy [5]. With an ever-ageing population, prognosticating “frailty” and organ failures has become extremely difficult due to fluctuating function with medical advancements [6]. Recent developments in cancer therapies mean that cancers previously considered to be terminal illnesses have become curable, or manageable long term if patients can effectively survive to receive full oncological treatment. These factors contribute to some of the many recognised difficulties seen by healthcare professionals in implementing effective EOLC in hospitals. This limited recognition of impending death is likely to have resulted in some patients in their final hours and days not receiving optimal care and perhaps even experiencing unnecessary pain, loss of dignity, and respect [2,7].

### 1.2. Palliative Care Medicine

Palliative Care Medicine (PCM) emerged from the hospice movement of the 1970s-80s, recognising the need for specialised care in terminal illness [2]. The World Health Organisation (WHO) defined palliative care (PC) in 1990, revising it in 2002. However, this was deemed too narrow, focusing only on life-threatening illness [8]. In 2018, the International Association for Hospice and Palliative Care (IAHPC) updated the definition to emphasise holistic, quality-of-life-focused care for patients and families: “Palliative care is the active holistic care of individuals across all ages with serious health-related suffering due to severe illness and especially of those near the end of life. It aims to improve the quality of life of patients, their families and their caregivers.” (Supplementary Material S1).

In the UK, guidance to improve EOLC include the Department of Health’s 2008 EOLC strategy document, advocating “a good death”, which includes being treated with dignity, free from pain, in familiar surroundings, and amongst loved ones [9]. The 2021–2026 “Ambitions for Palliative and End of Life Care”, a national framework produced by NHS England, aims to enhance local PC services by outlining six key aspirations to enhance palliative and end-of-life care across England [10].

The increasing medicalisation of death has contributed to a societal reluctance to engage with dying, mirrored by the limited visibility of palliative and end-of-life care within medical practice [11]. Often seen as end-of-life only, PC also benefits patients with chronic illness through symptom relief and support alongside curative treatment. As curative options decline, PC shifts to a multidisciplinary approach to comfort care [12]; however, despite its value, PC remains an underuse service.

### 1.3. Palliative Care in Intensive Care Units

Intensive Care Units (ICU) provide intensive monitoring and treatment for critically ill patients. Historically reserved for previously healthy individuals with acute conditions, ICU admissions now include more patients with complex, pre-existing illnesses. Approximately 20% of UK ICU admissions involve patients with severe and life limiting comorbidities [13]. The ICU environment may be distressing for both patients and families. Many patients arrive as emergencies, leaving little time for discussions about EOLC. Approximately 15% of ICU patients die during admission, up to 75% report distressing symptoms [14], and one in five survivors die within a year of discharge [15]. Despite this, PC is often introduced too late, or not at all, as ICU teams focus on life-sustaining treatments. When treatment escalation plans are considered, more than 80% of ICU patients lack the capacity to make decisions and only a minority of such patients have expressed anticipatory wishes. Families are often left to navigate these difficult choices, adding emotional burden.

Researchers have identified the merits and value of objective assessment tools to identify patients who may benefit from PC interventions early in their ICU admission. Several clinical decision-making tools exist to support clinical decision makers and provide helpful structures to streamline referrals to ensure timely intervention.

This study aims to evaluate the potential PC referral rates if a trigger tool was used on admission to the ICU and shows a positive uptake in referral rates with their use.

### 1.4. Aim and Objective

This study aims to evaluate the current practices of PC integration within a general ICU, with a specific focus on the utilisation and effectiveness of PC trigger tools. Our objectives are to determine the proportion of ICU patients who met criteria for PC referral based on established trigger tools and to compare the number of patients identified by trigger tools as appropriate for PC referral with the actual number of referrals made during the study period. We hope to identify potential gaps in the referral process and suggest strategies for improving PC integration in the ICU.

## 2. Materials and Methods

Institutional ethics approval was granted prior to the study. Extracted data were pseudonymised and securely stored on a password-protected system in accordance with General Data Protection regulation.

### 2.1. Setting

This study was conducted at a tertiary care institution in London and data were gathered from two general ICU wards, which comprised a total of 45 critical care beds, providing both level 2 (single-organ support/extensive post-op care) and level 3 (advanced respiratory or multi-organ support) care.

### 2.2. Cohort

All ICU deaths between 19 March and 30 October 2019 were included, with no exclusion criteria. These dates align with the introduction of the Electronic Healthcare Record (EHR) in March 2019, facilitating accurate data retrieval. October was selected as the endpoint to minimise the effects of the SARS-CoV-2 pandemic on ICU admissions.

### 2.3. Study Design 

The hospital EHR was used to identify all ICU deaths occurring in this period. Data collected included demographics, reason for ICU admission, length of ICU stay, whether a PC referral was made and the timing of referral relative to death. For those patients not referred to PC, three “trigger tools” were retrospectively and separately applied at the point of ICU admission:

- Hua et al. [16]—a five-point tool validated in ~400,000 ICU patients (USA).
- Zalenski et al. [17]—a five-point system from ~600 ICU patients (USA).
- Royal Marsden [RM] tool [18]—a seven-point consensus-based tool designed for oncology admissions (UK).

Patients were deemed suitable for PC referral if they met at least 1 criterion of the trigger tool being applied.

Table 1 sets out the criteria of each tool for referral to PC. The Hua et al. and Zalenski et al. tools were selected for their validation in large cohorts, with Hua et al. representing the most extensive study of its kind. The RM tool was included due to our study centre’s significant oncology caseload. The RM tool was only applied to those patients with an active cancer diagnosis.

### 2.4. Data Analysis

Descriptive statistics were employed to summarise the dataset. For categorical variables, absolute frequencies (n) and percentages (%) were calculated. Means and medians with ranges were used for continuous data.

Each patient record underwent individual examination. For patients who were referred to PC, the number of ICU days prior to referral and the interval from PC referral to death were recorded. Additional data collected included age, gender, type of ICU admission (planned or unplanned), underlying diagnosis, reason for ICU admission, cause of death, and total ICU length of stay (see Supplementary Materials S2).

For patients not referred to PC, clinical notes were reviewed against predefined trigger tools. Eligibility for PC referral was determined if any single criterion from each trigger tool was met at the time of ICU admission. No further statistical analyses were required for this study.

## 3. Results

### 3.1. Patient Demographics and Clinical Characteristics

Between March and October 2019, 121 patients died in the ICU. Table 2 outlines patient demographics. The mean age was 63 years (range: 21–92 years), with 59% (n = 71) being male. Planned admissions accounted for 8% (n = 10) of cases. Malignancy was the leading underlying diagnosis (49%, n = 59), followed by respiratory failure (17%, n = 21), sepsis (10%, n = 12), and neurological or surgical conditions.

### 3.2. Palliative Care Referrals

Table 3 outlines the PC referral details. Of the 121 patients, 28 (23%) received PC referrals, primarily for symptom control (96%, n = 27). Among these, 54% (n = 15) had an underlying malignancy. Notably, 36% (n = 10) of referred patients were not seen face-to-face by the PC team; in one case, telephone advice was provided. In 80% (n = 8) of cases where patients were not seen by PC, the referral occurred on the day of or the day before death.

### 3.3. Timing of Palliative Care Referrals

The mean ICU stay before referral was 7.5 days (range 0–29). The mean time from referral to death was 3 days (median 2 days; range 0–16, Table 4

### 3.4. Non-Referred Patients and Retrospective Trigger Tool Analysis

Overall, 93 patients (77%) were not referred to PC whilst in the ICU, although 5 of these had prior ward-based PC involvement. Of the 59 patients with an underlying malignancy, 75% (n = 44) were not referred to PC.

In total, 10 patients (8%) had prior PC involvement before ICU admission, but only 5 (<5%) were re-referred.

Retrospective application of the three trigger tools revealed that of the 93 patients not referred to PC, 75% (n = 70) of patients triggered at least one criterion using the Zalenski tool and 71% (n = 66) would have been eligible under the Hua et al. tool. Application of the RM tool showed 82% (n = 36) of cancer patients met referral criteria, (Table 5 and Table 6)

## 4. Discussion

Our study highlights a significant gap in palliative care provision, with up to 75% of ICU decedents meeting referral criteria but not being referred. Although the benefits of PC are well recognised, there appears to be a barrier to engage the service until the final day or days of illness, potentially limiting its effectiveness.

### 4.1. Barriers to Referral

One prevailing barrier to PC involvement in the ICU is the view that symptom management, often regarded as the core function of PC, is already well-addressed within the ICU setting by clinicians skilled in advanced supportive care [19]. Despite this, almost all the patients referred to PC in our study were referred for symptom control, indicating likely complex and refractory symptoms. Thirty-six percent of referred patients did not receive a face-to-face review, with the majority of those referred the day before or the day of death. This gives PC practitioners, who are already a stretched resource, limited time to review these complex patients but also at a time when the key benefits of symptom control are likely to be limited.

As society has moved towards improved healthcare and more medicalised, institutionalised death, western societies have become remote from discussions and experiences associated around death and dying [20]. This is reflected in the low profile of PC and EOLC, even within the medical profession. Palliative care remains a relatively new specialty and has historically received limited emphasis within medical curricula. It has often been associated primarily with end-of-life care in hospice settings. Consequently, many clinicians—particularly in intensive care units—may lack sufficient training, familiarity, and confidence in initiating PC consultations. This is amplified by the enduring belief that PC is synonymous with the withdrawal of curative efforts. As a result, PC is often only considered by ICU physicians once a poor prognosis is unequivocally established, due in part to concerns that early referral might be perceived as prematurely limiting active treatment.

Referral does not inevitably equate to limiting treatment options, but rather the early identification of those likely to benefit ensures patients and families have the time and support to make informed decisions. This can rarely be achieved on the day of or the day before death, yet the average ICU stay before referral in this study was 7.5 days.

Integrating PC necessitates not only addressing the patient’s medical needs but also actively involving their family. Families often experience significant emotional distress, confusion, and a sense of isolation when a loved one is admitted to the ICU. They may grapple with feelings of guilt or fear that consenting to palliative interventions equates to abandoning hope or failing their relative.

However, early and empathetic communication can alleviate these burdens. By engaging families, as well as patients, in discussions about the goals of care, clinicians can clarify that PC aims to enhance the quality of life, manage symptoms effectively, and support both the patient and their loved ones. This approach fosters shared decision-making, ensuring that care aligns with the patient’s values and the family’s expectations.

PC not only provides support for patients who are in the terminal stages of their illness but can also be employed to support those living with chronic and severe illnesses. Such patients often require specialised input for symptom control, support in making long-term decisions and achieving a good quality of life. This means that PC can in fact be employed in tandem with receiving care that is intended to cure underlying disease. When life-prolonging treatments become less effective, PC shifts toward intensive symptom management and psychosocial support [21]. Delivered by a multidisciplinary team, PC provides medical, emotional, and practical assistance but despite its broad relevance, PC remains underutilised due to misconceptions that it is reserved for imminent death.

### 4.2. Underlying Malignancy

For the deteriorating oncological patient, ICU admission is recognised as a valuable intervention but is typically reserved for patients with an anticipated good prognosis [22]. Despite this such patients often represent some of the sickest patients with a high risk of in-patient death. In this study, over half of PC referrals involved oncology patients, and retrospective application of the RM tool showed most malignancy-related deaths met at least one PC referral criterion. This reflects the hospital’s status as a specialised cancer centre where PC is well integrated into care pathways and yet remains challenging in ICU.

While traditionally associated with oncological services, PC is increasingly relevant across medical specialties as people live longer with chronic conditions [23]. Better integration of PC into hospital settings is crucial, particularly in ICUs, where mortality rates are comparatively high and predictable.

### 4.3. Trigger Tools

Most patients were not known to PC prior to admission to ICU, and 77% were not seen by PC before death, suggesting they were not expected to die either on the ward or in the ICU. This aligns with evidence that clinicians struggle to recognise dying patients [24], prioritising treatment trials before accepting death, or indeed, that these patients were not expected to die. While appropriate for reversible conditions, this highlights the need for trigger tools to aid in identifying patients who may benefit from early PC involvement to facilitate parallel planning which opens avenues for complementary care.

Although multiple trigger tools exist, they provide an objective framework to recognise dying patients. This study demonstrates that the use of a trigger tool would result in earlier PC referral. Early PC integration can improve quality of care, reduce unnecessary escalation of medical interventions, reduce hospital deaths, and enhance patient and family satisfaction.

The implementation of referral trigger tools can aid in systematically identifying patients who may benefit from palliative input, ensuring timely and appropriate involvement. However, the effectiveness of these tools is limited by the current capacity of palliative care teams. Despite the growing recognition of its value and widespread need, access to PC remains limited, with many services being underfunded and with a limited workforce capacity unable to meet the growing demand. As the need for palliative support continues to expand within critical care settings, greater investment for the integration of these services is essential to ensure equitable and high-quality care for all patients.

### 4.4. Limitations

This study’s findings are based on a relatively small cohort of 121 patients from two ICU wards within a single tertiary care institution in London. While this setting allowed for in-depth data collection and consistency in clinical practices, the limited sample size reduces the statistical power of the study and may affect the generalisability of the results to broader ICU populations. Consequently, the findings should be interpreted as preliminary and hypothesis-generating. Further research involving larger, more diverse patient cohorts across multiple centres is necessary to validate these results and enhance their applicability to varied clinical settings.

This study was conducted in a large teaching hospital with a high volume of oncology patients, also limiting generalisability. Additionally, two of the three trigger tools utilised in this study were developed within the context of US ICUs. Differences in healthcare delivery models between the US and UK—particularly around funding structures and referral pathways—may affect their applicability and implementation within the NHS.

The seven-month data collection period ended prior to the formal onset of the COVID-19 pandemic; nonetheless, early mortality trends related to the undetected spread of SARS-CoV-2 virus may have impacted the findings.

As a retrospective study, our findings are dependent on the accuracy and completeness of documentation within the EHR. At the time of data collection, the EHR system had been recently implemented, and it is possible that unfamiliarity with the new interface may have temporarily compromised documentation quality, leading to missing or incomplete data relevant to the study.

Some trigger tool criteria, such as “moderate or severe psychological distress,” are subjective, introducing potential interpretation bias. Additionally, defining “advanced or metastatic cancer” in haematological malignancies and patients receiving novel treatments may have proved challenging.

Despite these limitations, all three trigger tools identified an unmet need for PC referral. While this study did not assess the effectiveness of PC interventions, extensive evidence supports improved patient outcomes with early PC involvement. Importantly, we believe that valuable insights can be gained from examining the subset of patients who did receive early referral. Further work is warranted to elucidate the factors that contributed to timely referral in these cases and to identify best practices that may inform future care pathways.

## 5. Conclusions

The existence of multiple trigger tools underscores the complexity of real-world clinical decision-making, reflecting the clear need for such supporting frameworks. These tools offer an objective structure to aid in the recognition of dying patients and facilitate a ‘good death’.

This study demonstrates that the use of a trigger tool has the potential to increase PC referrals in the ICU. However, despite recognition of this approach as best practice, the findings reveal limited engagement with its implementation. It reinforces existing evidence supporting trigger tools in ICU settings and emphasises the evolving role of PC beyond end-of-life care, promoting a holistic, patient-centred approach. It is therefore imperative that in a patient population where mortality rates can be high and predictable, early PC involvement is considered. This shift has broad public health implications, fostering a less medicalised, more individualised approach to death, particularly in an ageing population, while encouraging open discussions on end-of-life planning.

Further research is needed to refine trigger tools for general ICU use, not only to identify those likely to die but also those who would benefit from PC in decision-making and long-term care. Crucially, studies must continue to gather evidence to establish whether increased PC consultation translates into tangible benefits for patients and families. In parallel, improving multidisciplinary team education in end-of-life and supportive care is essential to ensure that staff are equipped to recognise when PC input is appropriate and to deliver compassionate, coordinated care. PC remains an underfunded, resource-limited service, heavily reliant on charitable funding. Expanding PC access to ICUs will require significant investment, reinforcing the urgent need for increased funding to support this essential service.

## Figures and Tables

**Table 1:** Palliative care referral criteria on admission to ICU for three different trigger tools.

Palliative Care Trigger Tool	Hua et al. [16]	Zalenski et al. [17]	Royal Marsden [18]
	ICU admission after hospital stay >10 days	Advanced cancer	Metastatic cancer progressing after 1st line of treatment
**Criteria**	Stage IV malignancy	ICU stay >5 days or readmission within 30 days	Performance status ECOG 2 and deteriorating
	After cardiac arrest	After cardiac arrest	Acute oncology or unplanned admission
	ICH requiring ventilation	Team perceived the need for PC	Severe or overwhelming symptoms
	Multi-organ failure 3+ organ systems	Admitted from nursing facility	Anorexia, hypercalcaemia or any effusion
			Moderate or severe psychological distress
			Complex social issues

ICU, intensive care unit; ICH, intra-cerebral haemorrhage; PC, palliative care; ECOG, Eastern Cooperative Oncology Group. Comparison of palliative care referral triggers across three tools: Hua et al., Zalenski et al., and the Royal Marsden Hospital criteria. Each column outlines the criteria prompting consideration for palliative care consultation in the ICU setting.

**Table 2:** Patient demographics and clinical characteristics (N = 121).

Characteristic	Value
Mean age (years)	63 (21–92)
Male sex	71 (59%)
Planned admissions	10 (8%)
Underlying diagnosis:	
-Malignancy	59 (49%)
-Respiratory Failure	21 (17%)
-Sepsis	12 (10%)
-Neurological/surgical	29 (24%)

**Table 3:** Palliative care referral details (N = 28).

Variable	Value
Referral reason: symptom control	27 (96%)
Underlying malignancy	15 (54%)
Not seen face-to face by PC team	10 (36%)
Received telephone advice	1
Referral on the day of or day before death	8 (80% of those not seen)

**Table 4:** Timing of palliative care referrals.

Timing Metric	Value
Mean ICU stay before referral	7.5 days (range 0–29)
Mean time from referral to death	3 days (median 2, range 0–16)

**Table 5:** Retrospective trigger tool analysis for non-referred patients (N = 93).

Trigger Tool	Patients Meeting Criteria
Zalenski et al. tool	70 (75%)
Hua et al. tool	66 (71%)
RM tool	36 (82%)

**Table 6:** Retrospective trigger tool analysis for non-referred patients—number of patients meeting each criterion for palliative care referral on admission to ICU for each trigger tool.

Palliative Care Trigger Tool	Hua et al.		Zalenski et al.		Royal Marsden	
		n [%]		n [%]		n [%]
**Criteria**	ICU admission after hospital stay >10 days	31 [*33]	Advanced cancer	43 [*46]	Metastatic cancer progressing after 1st line of treatment	18 [†41]
	Stage IV malignancy	7 [*8]	ICU stay >5 days or readmission within 30 days	39 [*42]	Performance status ECOG 2 and deteriorating	6 [†14]
	After cardiac arrest	9 [*10]	After cardiac arrest	9 [*10]	Acute oncology or unplanned admission	26 [†59]
	ICH requiring ventilation	8 [*9]	Team perceived the need for PC	13 [*14]	Severe or overwhelming symptoms	20 [†45]
	Multi-organ failure 3+ organ systems	32 [*34]	Admitted from nursing facility	1 [*1]	Anorexia, hypercalcaemia, or any effusion	16 [†36]
					Moderate or severe psychological distress	5 [†11]
					Complex social issues	3 [†7]
**Number of patients who met any 1 criterion**		66 [*71]		70 [*75]		**36, [†82]**

Comparison of numbers of patients who met each criterion for each of the palliative care trigger tools Hua et al., Zalenski et al., and the Royal Marsden Hospital criteria on admission to ICU. ICU, intensive care unit; ICH, intra-cerebral haemorrhage; PC, palliative care; ECOG, Eastern Cooperative Oncology Group. * Percentages based on total number of patients who were not referred to PC. † Percentages based on total number of haematology/oncology patients who were not referred to PC (Supplementary Material S3).

## Data Availability

Data were extracted from patient notes and are displayed in Supplementary S2 anonymously.

## Supplementary Materials

The following supporting information can be downloaded at: https://www.mdpi.com/article/10.3390/jcm14124275/s1, S1: Palliative care definition from IAHPC in full; S2: Full dataset; S3: Patients who met trigger tool criteria.

## Abbreviations

The following abbreviations are used in this manuscript:

NHS	National Health Service
UK	United Kingdom
EOLC	End of Life Care
PCM	Palliative Care Medicine
WHO	World Health Organisation
PC	Palliative Care
IAHPC	International Association for Hospice and Palliative Care
DoH	Department of Health
ICU	Intensive Care Unit
UCLH	University College London Hospitals
A&E	Accident and Emergency department
RM	Royal Marsden Hospital
