# Supplementary material for: Utilising Palliative Care Expertise in Critically Ill Patients: Opportunities to Improve Outcomes and Experiences
Source: J Clin Med. 2025 Jun 16;14(12):4275. doi: 10.3390/jcm14124275 (PMC12194745; doi:10.3390/jcm14124275)
Supplement: Supplementary file 1 [file jcm-14-04275-s001.zip › jcm-3618756-supplementary.pdf]

Journal of Clinical Medicine

MDPI

Supplementary material 1: Palliative care definition from IAHPC in full

Palliative care is the active holistic care of individuals across all ages with serious health-related suffering[i] due to severe illness[ii], and especially of those near the end of life. It aims to improve the quality of life of patients, their families and their caregivers.

Palliative care:

- Includes, prevention, early identification, comprehensive assessment and management of physical issues, including pain and other distressing symptoms, psychological distress, spiritual distress and social needs. Whenever possible, these interventions must be evidence based.
- Provides support to help patients live as fully as possible until death by facilitating effective communication, helping them and their families determine goals of care.
- Is applicable throughout the course of an illness, according to the patient's needs.
- Is provided in conjunction with disease modifying therapies whenever needed.#May positively influence the course of illness.
- Intends neither to hasten nor postpone death, affirms life, and recognizes dying as a natural process.
- Provides support to the family and the caregivers during the patient's illness, and in their own bereavement.
- Is delivered recognizing and respecting the cultural values and beliefs of the patient and the family.
- Is applicable throughout all health care settings [place of residence and institutions] and in all levels [primary to tertiary].
- Can be provided by professionals with basic palliative care training.
- Requires specialist palliative care with a multiprofessional team for referral of complex cases.

To achieve palliative care integration, governments should:

1. Adopt adequate policies and norms that include palliative care in health laws, national health programs and national health budgets;
2. Ensure that insurance plans integrate palliative care as a component of programs;
3. Ensure access to essential medicines and technologies for pain relief and palliative care, including pediatric formulations;
4. Ensure that palliative care is part of all health services [from community health-based programs to hospitals], that everyone is assessed, and that all staff can provide basic palliative care with specialist teams available for referral and consultation;
5. Ensure access to adequate palliative care for vulnerable groups, including children and older persons;
6. Engage with universities, academia and teaching hospitals to include palliative care research as well as palliative care training as an integral component of ongoing education, including basic, intermediate, specialist, and continuing education.

[i] Suffering is health-related when it is associated with illness or injury of any kind. Health related suffering is serious when it cannot be relieved without professional intervention and when it compromises physical, social, spiritual and/or emotional functioning.

Supplementary material 2: Full data set

assigned r	age (years	gender	Underlyin	reason for	ICU days b	cause of d	ref/discus	known to	reason for	ICU days b	f2f r/v?	planned ic	days from	p/c refera	marsden-l	Zalenski-h	Hua-how r
2	34	F	follicular l	HAP/rising	11	PCP +/- VI	NO	no	n/a	n/a	n/a	unplanned			3	1	2
3	34	M	AML	resp failur	25	resp failur	YES	YES	symptom	not seen	yes	unplanne	45 days but on ward not ICU				
4	60	Female	cirrhosis a	low gcs/t2	1	resp failur	NO	NO	n/a	n/a	n/a	unplanne	n/a			1	1
5	34	m	burkitts ly	type 1 res	18	multi orga	YES	NO	symptom	17	YES	unplanne	0 days				
6	67	Female	DLBCL	low gcs	8		no	yes	symptom	n/a	no	unplanne	27 days		5	3	1
7	37	Female	aplastic ar	t1rf	2	neutroper	no	no	n/a	n/a	n/a	unplanne	n/a		2	1	1
8	79	Male	Decompe	Decompe	10	t2rf	no	no	n/a	n/a	n/a	unplanne	n/a				
9	72	Male	AML	deteriorat	3	?SEPSIS	no	no	n/a	n/a	n/a	unplanne	n/a		2	1	1
10	74	Male	lung ca	post thora	5	unclear	no	no	n/a	n/a	n/a	planned f	n/a			2	
11	77	Male	bilateral P	post thror	2	DIC	no	no	n/a	n/a	n/a	unplanne	N/A			1	2
12	36	Male	T cell lymp	type 1 res	12	MOF	no	no	n/a	n/a	n/a	unplanne	n/a		3	2	
13	64	Female	lung ca	post-op lc	8	resp failur	no	no	n/a	n/a	n/a	planned p	n/a			1	1
14	68	Female	endometr	t1rf	4	resp failur	yes	yes	symptom	2	yes	unplanne	8days				
15	65	Male	pancreatit	fluid man	31	sepsis	no	no	n/a	n/a	n/a	unplanne	n/a			1	1
16	76	Female	DLBCL	neutroper	1	sepsis	no	no	n/a	n/a	n/a	unplanne	n/a		4	1	1
17	75	Female	lung ca	resp failur	18	resp failur	no	no	n/a	n/a	n/a	unplanne	n/a		1	2	1
18	50	Male	likely lung	post- GI bl	2	HAP	yes	no	symptom	1	no	unplanne	1 day				
19	47	Male	T cell lymp	transfer fr	9	neutroper	no	no	n/a	n/a	n/a	planned f	n/a		1	3	2
20	65	Female	dlcbl	CAP/AKI	3	sepsis	no	no	n/a	n/a	n/a	unplanne	n/a		4	1	
21	77	Female	post op lo	post op lo	3	MOF seco	no	no	n/a	n/a	n/a	planned	n/a			1	
22	28	Female	B-ALL	low gcs pc	31	mof	no	yes	symptom	n/a	n/a	unplanne	65 days		3	2	2
23	41	Male	primary b	status epi	1	?aspiratio	yes	yes	symptom	1	yes	unplanne	in icu- 1				
24	52	Male	dlcbcl	low gcs	11	resp failur	no	no	n/a	n/a	n/a	unplanne	n/a		2	2	1
25	77	Female	post-op ol	post op cc	30	VAP	yes	no		29	1	unplanne	1 Day				
26	69	Male	AML	seizures	3	MOF	no	no	n/a	n/a	n/a	unplanne	n/a			1	1
27	65	Male	DLBCL	neutroper	33	hap	yes	no		28	no	unplanne	not seen 5 days				
28	68	Female	lung ca	post op V/	10	resp failur	no	no	n/a	n/a	n/a	planned	n/a		3	1	1
29	59	Female	lung ca	hypotensi	1	PE's	yes	no	symptom	1	no	unplanne	1 day				
30	83	Female	sepsis	hypotensi	2	sepsis	no	no	n/a	n/a	n/a	unplanne	n/a				1

assigned no	age (years)	gender	Underlying diagnosis	reason for ICU admissi	ICU days before death	cause of death	ref/discussed with PC on ICU?	known to PC	reason for pal care ref	ICU days before PC ref	f2f r/v?	planned icu	days from p/c referall to death	marsden-how many criteria triggered	Zalenski-how many criteria tr	Hua-how many criteria triggered
31	38	Female	asthma	collapse	3	asthma	no	no	n/a	n/a	n/a	unplanned	n/a		1	2
32	80	Male	liver disease	renal failure	19	liver/renal failure	yes	no	symptom	16	yes	unplanned	3 days			
33	42	Male	anaplastic lymphoma	cardiogenic shock	1	Cardiogenic shock & multi	no	no	n/a	n/a	n/a	unplanned	n/a	1	1	1
34	78	Male	Interstitial lung disease	t1rf	3	t1rf	yes	no	symptom	1	no	unplanned	2 days			
35	65	Male	tongue ca	transfer from another	4	septic shock	no	no	n/a	n/a	n/a	unplanned	n/a	2	1	
36	72	Male	heamorrhagic stroke	collaspe	3	heamorrhagic stroke	no	no	n/a	n/a	n/a	unplanned	n/a			1
37	64	Female	STEMI DLBCL	STEMI	20	resp failure	yes	no	symptom	6	yes	unplanned	14 days			
38	67	Male	lung ca	t1rf	9	resp failure	no	no	n/a	n/a	n/a	unplanned	n/a	5	2	1
39	69	Male	Oesophageal ca	neutropenic sepsis	4	resp failure	NO	no	n/a	n/a	n/a	unplanned	n/a	2	2	1
40	86	Male	heart failure	resp failure	4	resp failure	NO	no	n/a	n/a	n/a	unplanned	n/a		1	
41	58	Female	HLH	PUO	23	septic shock	no	no	n/a	n/a	n/a	unplanned	n/a		1	1
42	48	Male	DLBCL	HLH	2	MOF	no	no	n/a	n/a	n/a	planned	n/a	2	2	2
43	55	Male	CML	neutropenic sepsis	21	MOF	NO	no	n/a	n/a	n/a	unplanned	n/a	1	2	
44	77	Male	lung ca	t2rf	1	t2rf	NO	no	n/a	n/a	n/a	unplanned	n/a	3	2	1
45	63	Female	amyloidosis	SOB	14	heart failure	NO	no	n/a	n/a	n/a	unplanned	n/a		1	2
46	47	Male	Intracerebral heamorrhage	headache	2	intracerebral heamorrhage	NO	no	n/a	n/a	n/a	unplanned	n/a			2
47	75	Male	heart failure	SOB	4	heart failure	yes	no	symptom control	3	no	unplanned	1 day			
48	58	Male	Infective endocarditis	hyponatreamia	7	mof	no	no	n/a	n/a	n/a	unplanned	n/a		1	
49	71	Female	bowel ischeamia	abdo pain	2	bowel ischeamia and hear	no	no	n/a	n/a	n/a	unplanned	n/a		1	
50	21	Male	cervical spinal cord tumour	tetraplegia and resp fa	3	resp failure	yes	no	FAMILY	3	yes	unplanned	0 days			
51	72	Female	urosepsis	urosepsis-hypotension	1	mof	NO	no	n/a	N/A	n/a	unplanned	n/a			
52	87	Female	post-op laparatomy for perfo	sob	0	pul oedema	no	no	n/a	n/a	n/a	unplanned	n/a		1	
53	26	Female	brain tumour	headache	1	brain tumour	NO	NO	n/a	n/a	n/a	unplanned	n/a	3	1	
54	49	Male	ALL	sepsis	16	resp failure	no	yes	symptoms	n/a	n/a	unplanned	28	3	2	1
55	23	Male	ALL	neutropenic sepsis	8	mof	no	no	n/a	n/a	n/a	unplanned	n/a	3	2	2
56	21	Male	ALL	resp failure	7	chest sepsis	no	no	n/a	n/a	n/a	unplanned	n/a	2	2	1
57	71	Female	urosepsis	upper gi bleed and car	1	mof	no	no	n/a	n/a	n/a	unplanned	n/a		1	3
58	73	Female	AML	Resp failure	11	hap	no	no	n/a	n/a	n/a	unplanned	n/a	3	2	1
59	83	Male	post turp	cardiogenic shock	6	mof	no	no	n/a	n/a	n/a	unplanned	n/a		1	2
60	82	Female	cardiac arrest	cardiac arrest	3	resp failure	no	no	n/a	n/a	n/a	unplanned	n/a		1	1

assigned no	age (years)	gender	Underlying diagnosis	reason for ICU admission	ICU days before death	cause of death	ref/discussed with PC on ICU?	known to PC	reason for pal care ref	ICU days before PC ref	f2f r/v?	planned icu vs unplann	days from p/c referall to death	marsden-how many criteria triggered	Zalenski-how many criteri	Hua-how many criteria triggered
61	58	Female	met lung ca	resp failure	3	resp failure	yes	yes	symptom	0	yes	unplanned	16 days			
62	72	Male	post-op perforation	septic- feacal peritonitis	2	mof	no	no	n/a	n/a	n/a	planned	n/a			1
63	65	Male	multiple brain infarcts	low gcs post thrombolysis	1	brain infarcts	no	no	n/a	n/a	n/a	unplanned	n/a		1	
64	62	Female	idiopathic pumonary fib	low oxygen levels	1	resp failure	no	yes	symptom	n/a	n/a	unplanned	10		2	1
65	83	Male	lung ca	t2rf	2	HAP	no	no	n/a	n/a	n/a	unplanned	n/a	4	2	
66	75	Female	brain heamorrhage	collapse	1	brain heamorrgahe	no	no	n/a	n/a	n/a	unplanned	n/a			1
67	60	Female	neutropenic sepsis	resp failure	11	unknown	yes	no	symptom	9	yes	unplanned	2 days			
68	60	Male	HAP lung ca	resp failure/gi bleed	15	HAP	no	no	n/a	n/a	n/a	unplnned	n/a		2	
69	66	Male	DLBCL	aki/ali	2	mof	no	no	n/a	n/a	n/a	unplanned	n/a	1	1	
70	84	Male	sepsis	aki	2	mof	no	no	n/a	n/a	n/a	unplanned	n/a			
71	89	Male	pneumonia	resp failure	10	resp failure	no	no	n/a	n/a	n/a	unplanned	n/a		1	1
72	63	Male	recurrent pneumonia	aspiration pneumonia	19	resp failure	yes	no	symptom	11	yes	unplanned	6 days			
73	58	Male	AML	chest sepsis	9	resp failure	yes	no	symptom	8	no	unplanned	1 day			
74	73	Male	mof	low gcs	0	mof	no	no	n/a	n/a	n/a	unplanned	n/a		2	2
75	67	Male	tls /follicular lymphoma	aki/pul oedema	2	renal failure	no	no	n/a	n/a	n/a	unplanned	n/a	3	1	
76	56	Male	PE	cardiac arrest	10	mof	no	no	n/a	n/a	n/a	unplanned	n/a		2	1
77	90	Male	sepsis	Sepsis	13	HAP	yes	no	symptom	8	yes	unplanned	7 days			
78	71	Female	tcell lymphoma	neutropenic sepsis/resp failu	3	mof	no	no	n/a	n/a	n/a	unplanned	n/a	2	1	1
79	34	Female	AML	Resp failure	25	mof	no	no	n/a	n/a	n/a	unplanned	n/a	3	2	1
80	34	Male	cardiac arrest	hypoxic brain injury cardiac a	7	hypoxic brain injury	no	no	n/a	n/a	n/a	unplanned	n/a		2	1
81	71	Male	pancreatitis	pancreatitis/AKI	3	acute pancreatitis	no	no	n/a	n/a	n/a	unplanned	n/a			
82	76	Male	AML	T1RF HAP	25	resp failure	no	no	n/a	n/a	n/a	unplanned	n/a	3	3	1
83	84	Male	mof	collapse	1	mof	no	no	n/a	n/a	n/a	unplanned	n/a			1
84	69	Female	AML	neutropenic sepsis	7	mof	no	no	n/a	n/a	n/a	unplanned	n/a	3	3	1
85	61	Male	pancreatitis	sepsis	2	mof	no	no	n/a	n/a	n/a	unplanned	n/a			1
86	64	Female	gi tumour- unknown	collapse	1	massive heamorrhage	no	no	n/a	n/a	n/a	unplanned	n/a		1	
87	92	Female	Intracerebral heamorrha	low gcs	1	intracerebral heamorr	no	no	n/a	n/a	n/a	unplanned	n/a			1
88	62	Female	paracetamol overdose	ALF	3	aspiration pneumonia	no	no	n/a	n/a	n/a	unplanned	n/a			
89	53	Female	sepsis	seizures	7	mof	no	no	n/a	n/a	n/a	unplanned	n/a		1	1
90	69	Male	pneumonitis	resp failure	6	resp failure	yes	no	symptom control	5	no	unplanned	1 day			

assigned no	age (years)	gender	Underlying diagnosis	reason for ICU admission	ICU days before death	cause of death	ref/discussed with PC on ICU?	known to PC	reason for pal care ref	ICU days before PC ref	f2f r/v?	planned icu vs u	days from p/c referall to death	marsden-how m	Zalenski-how many	Hua-how many criteria triggered
91	54	Female	chest sepsis	sepsis	5	mof	no	no	n/a	n/a	n/a	unplanned	n/a			
92	46	Female	LRTI cardiac arrest	cardiac arrest	3	mof	no	no	n/a	n/a	n/a	unplanned	n/a		1	2
93	71	Male	urosepsis	septic shock	2	sepsis	no	no	n/a	n/a	n/a	unplanned	n/a			1
94	60	Male	follicular lymphoma	t1rf	19	mof	no	no	n/a	n/a	n/a	unplaned	n/a	3	2	1
95	75	Male	anastomotic leak	post-op emergency laparot	11	pulmonary heamorrh	no	no	n/a	n/a	n/a	unplanned	n/a		1	1
96	65	Male	AML	resp failure/AKI	2	resp sepsis	yes	YES	symptom	0	yes	unplanned	4 days			
97	76	Male	gi tumour	gi bleeding	3	mof	no	no	n/a	n/a	n/a	unplanned	n/a	3	2	
98	66	Female	ovarian ca	aki	1	mof	no	no	n/a	n/a	n/a	unplanned	n/a	2	2	3
99	19	Female	post cardiac arrest	post cardiac arrest	6	hypoxic brain injury	no	no	n/a	n/a	n/a	unplanned	n/a		3	1
100	88	Female	unclear	aki	3	unclear- mof	no	no	n/a	n/a	n/a	unplanned	n/a			2
101	66	Female	AML	T1RF	26	intracerebral heamo	yes	no	symptom	24	yes	unplanned	3 days			
102	79	Male	Intracerebral heamorrha	collapse	1	intracerebral heamo	no	no	n/a	n/a	n/a	unplanned	n/a			1
103	80	Male	pneumonia and kidney d	AKI/Low GCS	5	pneumonia	no	yes	symptoms	n/a	yes	unplanned	5		1	1
104	83	Male	Intracerebral heamorrha	collapse	3	intracerebral heamo	no	no	n/a	n/a	n/a	unplanned	n/a			1
105	38	Male	pneumonia	t2rf	4	t2rf	yes	no	symptom	1	yes	unplanned	3days			
106	43	Female	pneumonia	resp failure	29	mof	no	no	n/a	n/a	n/a	unplanned	n/a		1	
107	85	Male	nstemi	post cardiac arrest	7	resp failure	yes	no	symtom	3	yes	unplanned	4 days			
108	50	Female	melanoma	septic	2	AKI	yes	no	symptom	1	no	unplanned	0 days			
109	54	Male	Intracerebral heamorrha	collaspe	2	intracerebral heamo	no	no	n/a	n/a	n/a	unplanned	n/a			1
110	83	Male	bowel ischeamia	bowel ischeamia	1	bowel ischeamia	no	no	n/a	n/a	n/a	unplanned	n/a		1	1
111	74	Male	lrti/pulmonary fibrosis	resp failure	1	lrti	yes	no	symptom	0	yes	unplanned	0 days			
112	80	Male	t2rf	post-op VATS	7	t2rf	yes	no	symptom	4	yes	planned	3 days			
113	67	Female	Intracerebral heamorrha	collapse	2	intracerebral heamo	no	no	n/a	n/a	n/a	unplanned	n/a			1
114	25	Male	burkitts lymphoma	tumour lysis syndrome	4	mof	no	no	n/a	n/a	n/a	unplanned	n/a	1	1	2
115	85	Male	hap	septic	1	hap	no	no	n/a	n/a	n/a	unplanned	n/a			1
116	72	Female	acute leukeamia	resp failure	18	intracerebral heamo	yes	no	symptom	18	no	unplanned	0 days			
117	60	Female	sepsis	neutropenic sepsis	2	mof	no	no	n/a	n/a	n/a	unplanned	n/a		1	2
118	53	Male	metastatic cholangiocar	mof	1	mof	no	no	n/a	n/a	n/a	unplannned	n/a	3	1	2
119	53	Male	cap sec to pulmonary fib	CAP	3	resp failure	no	no	n/a	n/a	n/a	unplanned	n/a			
120	44	Female	ttp	cardiac arrest	0	cardiac arrest	no	no	n/a	n/a	n/a	planned	n/a			
121	60	Female	septic	septic shock	5	mof	yes	no	symptom	4	no	unplanned	1 days			
122	88	Male	post laparotomy	post laparotomy	1	complete heart block	no	no	n/a	n/a	n/a	planned	n/a		1	1
	#REF!				#REF!					#REF!						
														36 PATIENTS	70 PATIENTS	66 PATIENTS

Supplementary material 3: Patients who met trigger tool criteria

Marsden - CANCER pts only	
METCa progessing after 1st line treatment	2, 6, 12, 16, 20, 22, 28, 38, 42, 53, 54, 55, 58, 65, 75, 84, 94, 98
performance status ECOG 2 and deteriorating - ambulat	6, 16, 38, 65, 84, 97
acute oncology or unplanned admission (to hospital)	2, 6, 7, 9, 12, 16, 17, 20, 22, 24, 28, 33, 35, 38, 39, 42, 43, 44, 53, 55, 56, 58, 65, 69, 97, 118
severe or overwhealming symptoms SUBJECTIVE/WHEN	2, 7, 9, 19, 20, 22, 28, 35, 38, 44, 53, 65, 75, 78, 79, 82, 94, 97, 114, 118
anorexia, high ca, any effusion	6, 12, 16, 20, 38, 39, 44, 55, 56, 75, 78, 79, 82, 84, 94, 98
mod or severe phsycho or existential distress	6, 54, 58, 79, 82
complex social issues	24, 54, 118
Zalenski - MEDICAL ICU	
(Advanced) ca	2, 6, 7, 9, 10, 12, 13, 16, 17, 19, 20, 21, 22, 24, 26, 33, 35, 38, 39, 42, 43, 44, 53, 54, 55, 56, 58, 65, 68, 69, 75, 78, 79, 82, 84, 86, 94, 97, 98, 110, 114, 117,
icu stay over 5 days or readmission within 30 days	4, 6, 10, 12, 13, 15, 17, 19, 22, 24, 28, 38, 41, 43, 44, 45, 48, 52, 54, 55, 56, 58, 59, 64, 65, 68, 71, 74 76, 79, 80, 82, 84, 89, 94, 95, 99, 106, 122
admission post-arrest	11, 31, 57, 60, 74, 76, 80, 92, 99
team percieved need PC	6, 19, 39, 40, 42, 49, 63, 64, 82, 84, 97, 98, 103
Admitted from a nursing facility	99
Hua et al- adapted from elsewhere	
icu admission after a hospital stay of 10 or more days	2, 4, 6, 7, 13, 15, 16, 17, 19, 22, 24, 26, 41, 42, 45, 54, 55, 56, 57, 58, 59, 64, 71, 78, 82, 84, 94, 98, 100, 103, 110
diagnosis of stage 4 malignancy (metastatic) EXCLUDES I	28, 38, 44, 98, 114, 117, 118
post CA	11, 31, 57, 60, 74, 76, 80, 92, 99
intracerebral heamorrhage requiring ventilation	36, 46, 66, 87, 102, 104, 109, 113
multisystem organ failure of 3+ systems (during ICU)	2, 9, 11, 19, 22, 30, 31, 33, 39, 42, 45, 46, 55, 57, 59, 62, 74. 76, 79, 83, 85, 89, 92, 93, 95, 98, 100, 114, 115, 117, 118, 122
